# Small intestinal immune-environmental changes induced by oral tolerance inhibit experimental atopic dermatitis

**DOI:** 10.1038/s41419-021-03534-w

**Published:** 2021-03-04

**Authors:** Han-Na Um, Jin-Ok Baek, Sohyeon Park, Eun-Hui Lee, Jinsun Jang, Woo-Jae Park, Joo-Young Roh, YunJae Jung

**Affiliations:** 1 Department of Health Science and Technology, Gachon Advanced Institute for Health Science & Technology, Gachon University, Incheon, 21999 South Korea; 2 Department of Dermatology, Gachon Gil Medical Center, College of Medicine, Gachon University, Incheon, 21565 Korea; 3 Department of Microbiology, College of Medicine, Gachon University, Incheon, 21999 Korea; 4 Department of Biochemistry, College of Medicine, Gachon University, Incheon, 21999 Korea

**Keywords:** Allergy, Mucosal immunology

## Abstract

Atopic dermatitis is a chronic skin inflammatory disease mediated by Th2-type immune responses. Although intestinal immune responses have been shown to play a critical role in the development or prevention of atopic dermatitis, the precise influence of intestinal immunity on atopic dermatitis is incompletely understood. We show here that orally tolerized mice are protected from experimental atopic dermatitis induced by sensitization and epicutaneous (EC) challenge to ovalbumin. Although the expression of Th2-type cytokines in the small intestine of orally tolerized and EC-challenged mice did not change significantly, these mice showed decreased inflammatory responses in the small intestine with restoration of microbial change elicited by the EC challenge. Interestingly, an increase in small intestinal eosinophils was observed with the EC challenge, which was also inhibited by oral tolerance. The role of small intestinal eosinophils and microbiota in the pathogenesis of experimental atopic dermatitis was further substantiated by decreased inflammatory mediators in the small intestine and attenuated Th2-type inflammation in the skin of eosinophil-deficient and microbiota-ablated mice with EC challenges. Based on these data, we propose that the bidirectional interaction between the skin and the intestine has a role in the pathogenesis of atopic dermatitis and that modulation of the intestinal microenvironments could be a therapeutic approach to atopic dermatitis.

## Introduction

Atopic dermatitis (AD) is a chronic inflammatory skin disease characterized by persistent itching accompanied by pathological changes in the skin^1,2^. The prevalence of AD is considerably higher in developed countries than in developing countries, suggesting that genetic susceptibility and a dysregulated immune response to environmental antigens contribute to the global AD epidemic^3^. Mutations in genes that encode proteins involved in keratinocyte terminal differentiation, including filaggrin, loricrin, involucrin, small proline-rich proteins, S100A family proteins, and late cornified envelope proteins, are the most significant genetic risk factors for AD development^4,5^. In response to various environmental factors, such as skin irritants, climate, pollutants, tobacco smoke, water hardness, and diet^6^, the damaged epithelium of the skin releases innate inflammatory cytokines such as IL-25, IL-23, and thymic stromal lymphopoietin, which promotes an aberrant Th2-type inflammation by triggering the production of IL-4, IL-5, IL-13, IL-31, and IL-10 (ref. ^7,8^). Based on the proposition that a defective epithelial barrier has a pathogenic role in AD, a murine model of AD has been developed by the repeated epicutaneous (EC) exposure of tape-stripped skin to ovalbumin (OVA)^9,10^.

Food and inhaled allergens induce atopic skin lesions, which is also indicated by the pathogenic role of food allergies in AD development and the beneficial effects of elimination diets observed in children with AD^11,12^. Increasing evidence indicates that along with skin barrier dysfunction, intestinal permeability is also compromised in patients with AD^3,13^. An important concept that has emerged in this context is that a dysfunctional intestinal barrier is the key driver of AD, as exposure to allergens via the damaged intestinal mucosa causes oral tolerance to be bypassed^14,15^. Oral tolerance is the state of local and systemic immune unresponsiveness induced by orally ingested innocuous antigens^16^. Findings from our studies as well as other studies showed the prevention of murine AD-like inflammation mediated by oral tolerance^17,18^. Notably, the skin and intestine share anatomical properties^19^, and these tissues serve as the primary line of defense against exposure to the external environment. However, it is yet to be determined if homeostatic intestinal immune responses, represented by oral tolerance, play a role in protection from allergic skin inflammation.

In this study, using a murine AD model combined with oral tolerance induction, we investigated the role of the homeostatic intestinal immune response in protection against allergic skin inflammation. Based on our findings, we propose that the bidirectional interaction between cutaneous and intestinal immune responses plays a role in AD development, and that the promotion of intestinal immune homeostasis could ameliorate allergic manifestations in the skin.

## Results

### Oral tolerance induction inhibited AD-like inflammatory changes in the skin induced by sensitization and EC challenge with OVA

**Fig. 1:**
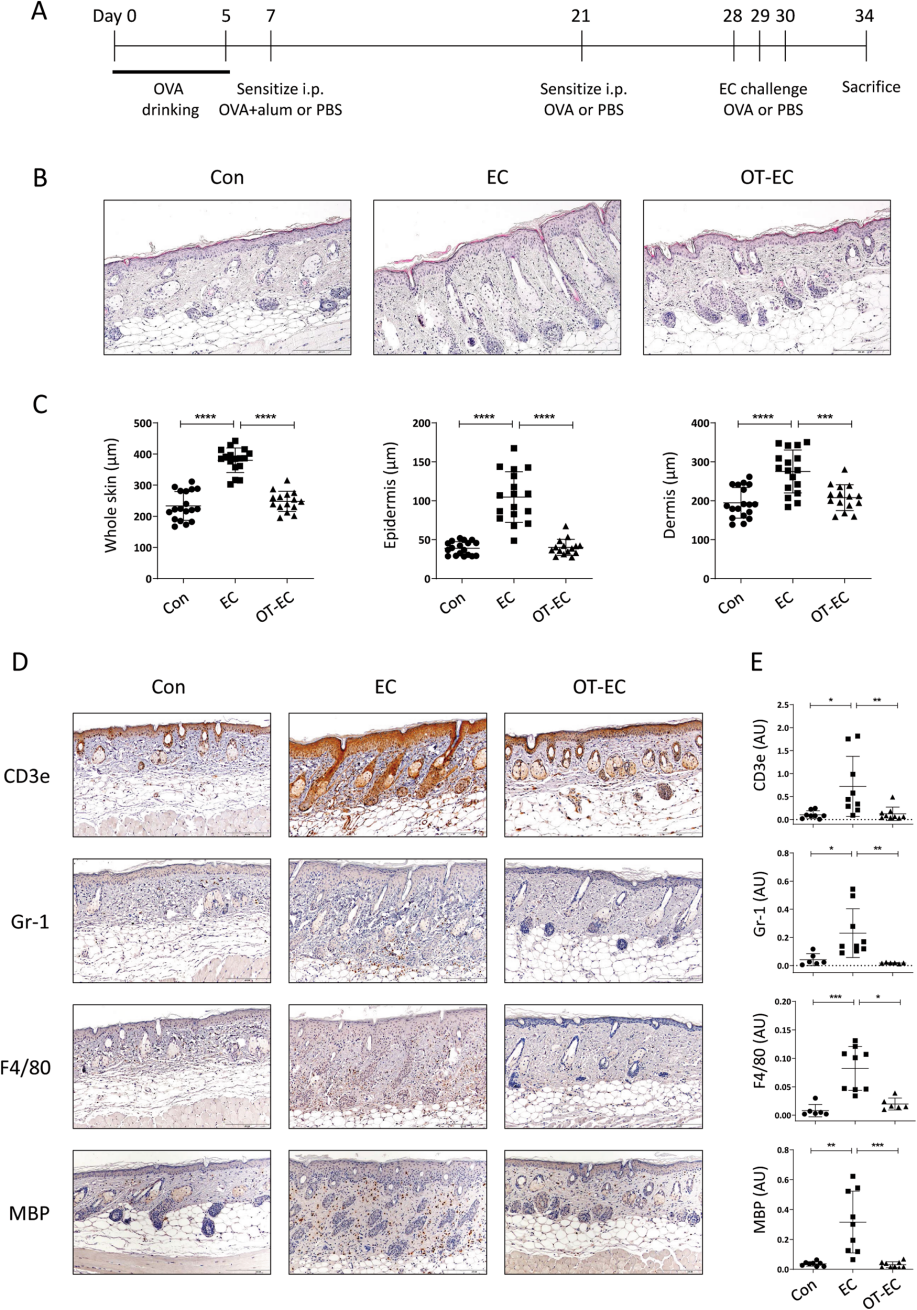
Histological inflammation and immune cell infiltration of the skin of EC-challenged mice was inhibited by oral tolerance induction. **A** Experimental protocol. Mice were fed with or without 1% OVA in drinking water for 5 days prior to sensitization with two intraperitoneal injections of OVA-alum or OVA, followed by three consecutive EC challenges with OVA. The control mice were sensitized with PBS followed by EC challenge with PBS. **B** Hematoxylin and eosin staining of skin from the control (Con), EC-challenged (EC), and orally tolerized and EC-challenged (OT-EC) mice. The images are representative of two independent experiments. The scale bar represents 200 μm. **C** The thickness of the whole skin (left), epidermis (middle), and dermis (right) of the indicated mice group. The graphs show the mean ± SD values. ***P < 0.001, ****P < 0.0001 (one-way ANOVA). **D** and **E** Immunohistochemical staining **D** and intensity quantification **E** of T cells (CD3e), neutrophils (Gr-1), macrophages (F4/80), and eosinophils (MBP) in the skin of the indicated mice. Images are representative of two independent experiments. The scale bar represents 200 μm. The graphs show the mean ± SD values. *P < 0.05, **P < 0.01, ***P < 0.001 (Kruskal–Wallis test).

**Fig. 2:**
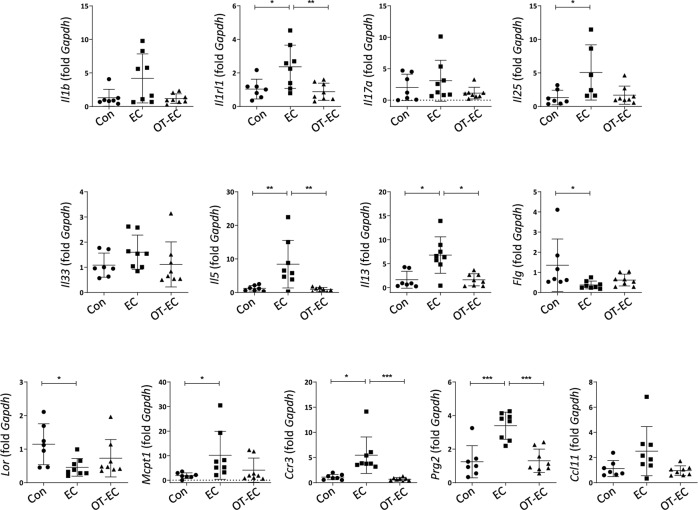
Skin expression of allergic inflammatory markers of EC-challenged mice was inhibited by oral tolerance induction. mRNA expression of *Il1b, Il1rl1*, *Il17a*, *Il25*, *Il33*, *Il5*, *Il13*, *Flg*, *Lor*, *Mcpt1*, *Ccr3*, *Prg2*, and *Ccl11* in the skin of the indicated mice. All data are representative of two independent experiments. The graphs show the mean ± SD values. *P < 0.05, **P < 0.01, ***P < 0.001 (one-way ANOVA for *Il1rl1*, *Il5*, *Flg*, and *Prg2*; Kruskal–Wallis test for *Il25*, *Il13*, *Lor*, *Mcpt1*, and *Ccr3*).

To explore the detailed immunological changes in atopic inflammatory skin with oral tolerance, we fed mice with OVA in drinking water, and then induced AD-like inflammation via repeated EC exposure to OVA, following systemic OVA sensitization (Fig. 1A). The epidermal and dermal thicknesses, which are associated with epidermal hyperplasia and dermal infiltration, increased in EC-challenged mice (Fig. 1B and C). However, in orally tolerized mice, histologically evident pathological changes were not observed in the skin, even with the subsequent EC challenge (Fig. 1B and C). In line with these findings, the infiltration of lymphoid (CD3e^+^ T cells) and myeloid (Gr-1^+^ neutrophils, F4/80^+^ macrophages, and MBP^+^ (major basic protein) eosinophil) cells observed in the skin of EC-challenged mice was inhibited significantly in orally tolerized mice (Fig. 1D and E). We then analyzed the expression of Th2-type inflammatory mediators in the skin of EC-challenged mice with or without prior induction of oral tolerance. As shown in Fig. 2, the expression of genes encoding innate cytokines, such as *Il1rl1* (gene encoding ST2, a receptor for IL-33) and *Il25*, which induce allergic inflammation^7,20^, increased significantly in the skin of EC-challenged mice, along with an increase in the expression of classical Th2-type cytokines (*Il5* and *Il13*) and a decrease in the expression of skin barrier markers (*Flg* and *Lor*). The expression of genes encoding inflammatory mediators associated with eosinophil infiltration and activation (*Ccr3* and *Prg2*) and mast cell activation (*Mcpt1*) also increased significantly in the skin of EC-challenged mice (Fig. 2). However, the upregulation of the listed Th2-type inflammatory markers was efficiently blocked in mice with oral tolerance, as indicated by the insignificant differences in the expression compared to that in mice in the control group (Fig. 2). Collectively, our observations indicate that the induction of oral tolerance to an antigen before EC exposure efficiently inhibited allergic skin inflammation induced upon EC challenge with the same antigen.

### Oral tolerance induction restored intestinal immune-environmental changes induced by EC challenge with OVA

**Fig. 3:**
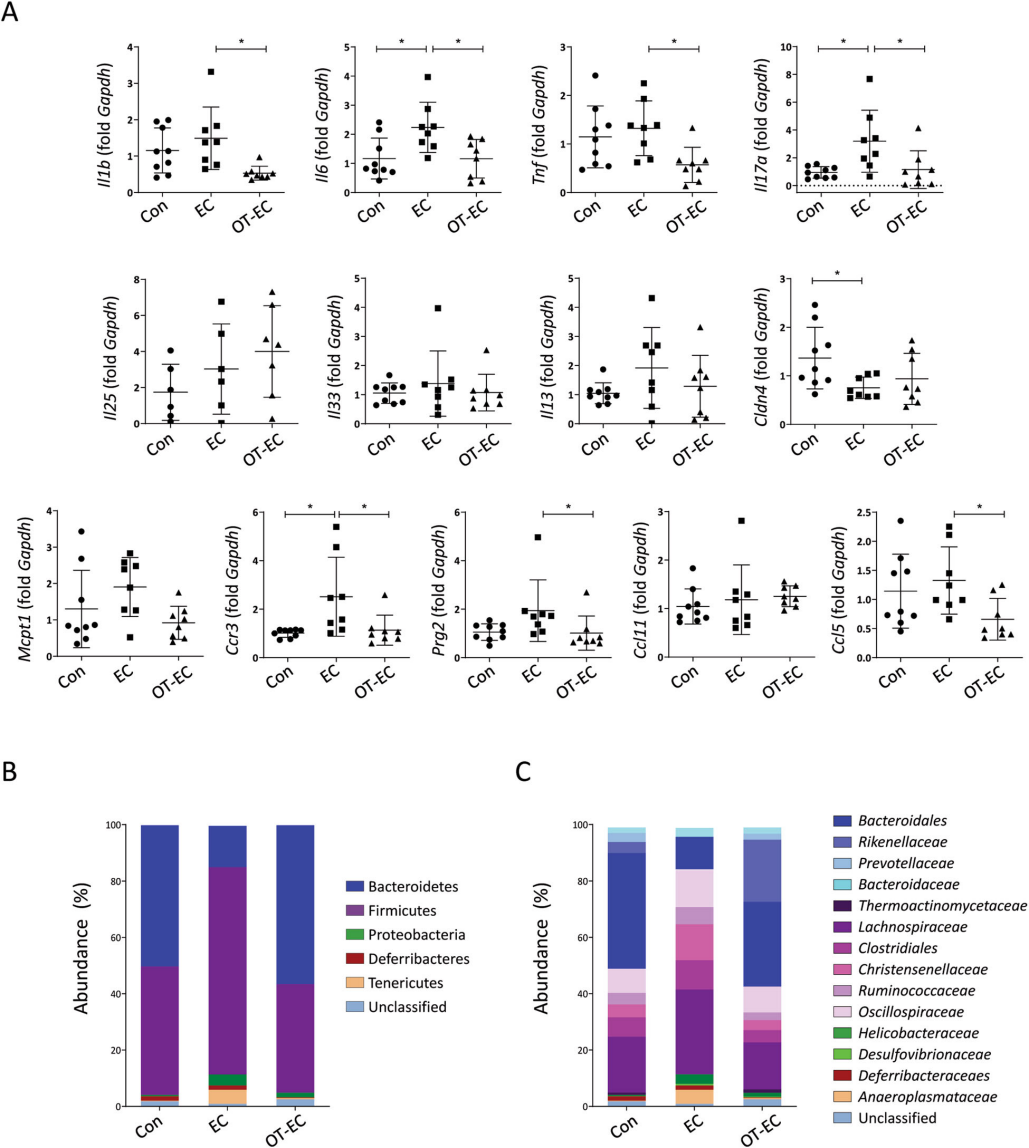
EC challenge and oral tolerance induction elicited immune microenvironmental changes in the small intestine. **A** mRNA expression of *Il1b*, *Il6, Tnf*, *Il17a*, *Il25*, *Il33*, *Il13*, *Cldn4*, *Mcpt1*, *Ccr3*, *Prg2*, *Ccl11*, and *Ccl5* in the small intestine of the indicated mice. All data are representative of two independent experiments. The graphs show the mean ± SD values. *P < 0.05 (one-way ANOVA for *Il1b*, *Tnf*, *Il17a*, and *Cldn4*; Kruskal–Wallis test for *Il6*, *Ccr3*, *Prg2*, and *Ccl5*). **B** and **C** shows relative abundance at the phylum **B** and family levels **C** of microbiota in the cecal contents of the indicated mice. Each column represents pooled samples from the indicated mice (*n* = 3 mice per group).

As the digestion and absorption of orally administered antigens occur only in the small intestine^21^, we hypothesized that changes in the small intestinal immune responses influence the oral tolerance-mediated inhibition of allergic skin inflammation. Therefore, we assessed whether EC exposure to OVA induces an increase in the expression of inflammatory mediators in the small intestine and whether oral tolerance is associated with an expressional change in these mediators in the small intestine of EC-challenged mice. As shown in Fig. 3A, a significant upregulation of *Il6*, *Il17a*, and *Ccr3* and downregulation of *Cldn4* (gene encoding epithelial tight junction claudin-4) mRNA were observed in the small intestine of EC-challenged mice, which were indicative of intestinal inflammatory changes induced by EC antigen challenge. The induction of oral tolerance inhibited the expression of these mediators and significantly suppressed the expression of *Il1b*, *Tnf*, *Prg2*, and *Ccl5* mRNAs in the small intestine compared to that in EC challenged mice. As the expression of *Il25*, *Il33*, *Il13*, and *Mcpt1* mRNAs in the small intestine was not significantly altered by either EC challenge or oral tolerance (Fig. 3A), it is unlikely that immune responses in the small intestine of EC-challenged mice are polarized into Th2-type inflammation. Aberrant immune responses in the intestine, which are often accompanied by the abnormal production of inflammatory cytokines, are linked to an imbalance in the intestinal microbiota^22^. To investigate the global shift in intestinal bacterial communities caused by EC challenge or oral tolerance induction, we performed 16S rRNA sequencing of fecal samples collected from the cecum of EC-challenged mice with or without oral tolerance. In principal coordinate analysis, where samples with similar microbial profiles are clustered together, the control and orally tolerized fecal samples were clustered, whereas samples from the EC-challenged mice clustered individually with the control and the orally tolerized samples (Fig. S1). At the phylum level, compared to that in the control group, a marked increase in Firmicutes (Mann–Whitney test, *P* = 0.0500) and Tenericutes (Mann–Whitney test, *P* = 0.0383) and a decrease in Bacteroidetes (Mann–Whitney test, *P* = 0.0500) were observed in EC-challenged mice, which was reversed in orally tolerized mice (Fig. 3B). At the family level, an increase in *Lachnospiraceae* (Mann–Whitney test, *P* = 0.0500) and *Christensenellaceae* (Mann–Whitney test, *P* = 0.0500) and a significant decrease in *Bacteroidales* (Mann–Whitney test, *P* = 0.0500) and *Prevotellaceae* (Mann–Whitney test, *P* = 0.0500) were observed in the EC-challenged mice compared to that in control group mice (Fig. 3C). The microbial composition of the control group mice and the orally tolerized mice did not differ significantly at the phylum and family levels (Fig. 3B and C).

### Oral tolerance induction inhibited the increase in small intestinal eosinophils induced upon EC challenge with OVA

**Fig. 4:**
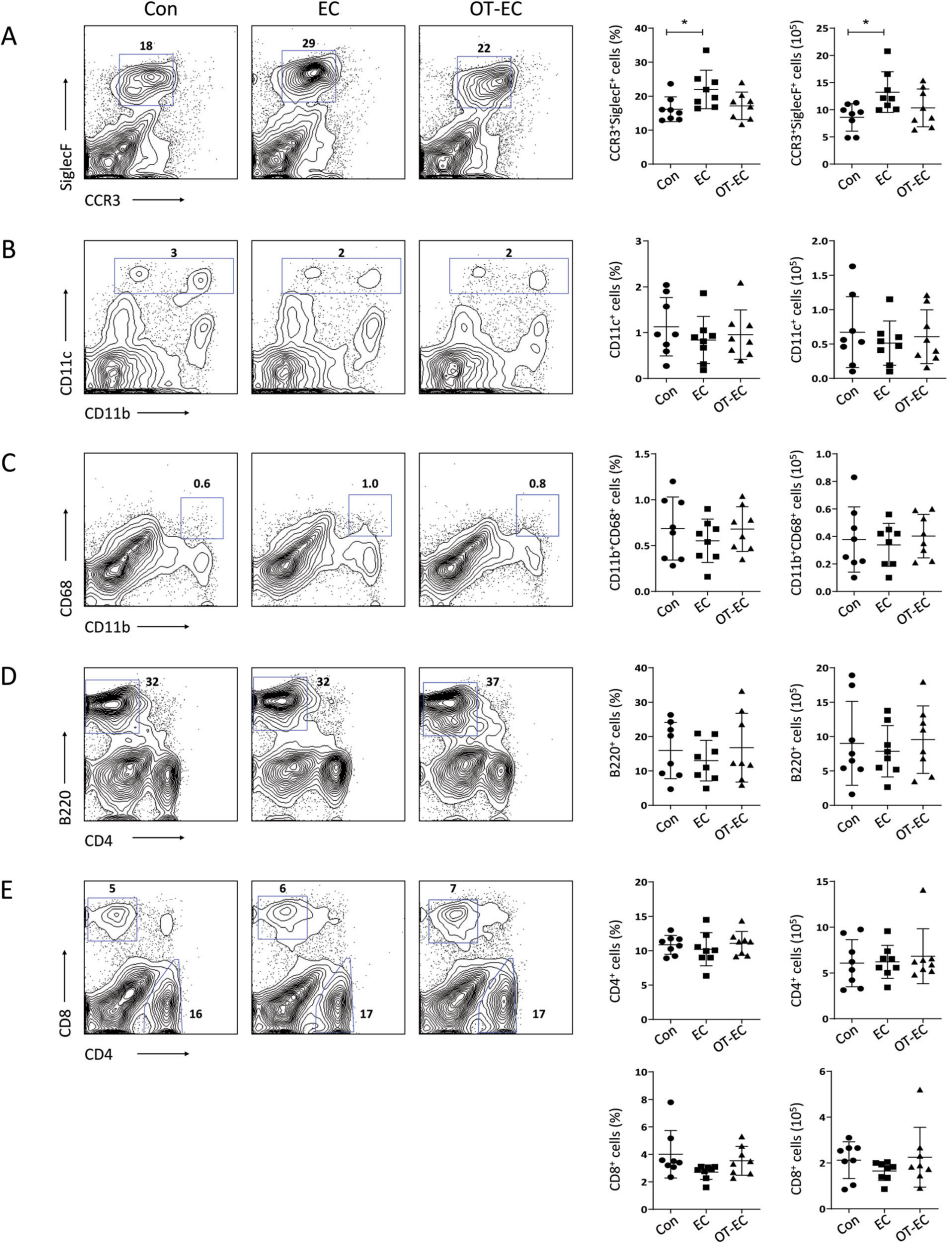
Oral tolerance inhibited the infiltration of the small intestinal eosinophils elicited by EC challenge. **A**–**E** Representative flow cytometric plots, percentage, and cell count of eosinophils **A**, dendritic cells **B**, macrophages **C**, B cells **D**, and CD4^+^ and CD8^+^ T cells **E** in the small intestine of the indicated mice. The plots are gated on CD45^+^ cells and are representative of two independent experiments. The graphs show the mean ± SD values. *P < 0.05 (one-way ANOVA).

The lamina propria of the small intestine contains multiple immune cells, including B cells, T cells, and several types of innate cells, such as eosinophils, macrophages, and dendritic cells, which orchestrate intestinal immune responses through active interaction with antigenic stimuli^21^. Therefore, we studied the immune cell populations in the lamina propria of the small intestine to identify cellular changes induced by EC challenge or oral tolerance. The total number of CD45^+^ leukocytes isolated from the small intestinal lamina propria of the control, EC-challenged, and orally tolerized mice did not differ significantly (Fig. S2). The abundance of CD4^+^ and CD8^+^ T cells, B220^+^ B cells, CD11b^+^CD68^+^ macrophages, and CD11c^+^ dendritic cells did not change significantly upon EC challenge or oral tolerance (Fig. 4B–E). Notably, the frequency and number of CCR3^+^SiglecF^+^ eosinophils increased significantly in the small intestine of EC-challenged mice (Fig. 4A), suggesting a bidirectional relationship between cutaneous and gastrointestinal eosinophils. The frequency and number of eosinophils in the small intestine of orally tolerized mice were comparable to those in control mice (Fig. 4A).

### Inflammatory changes in the skin and small intestine of OVA EC-challenged mice were inhibited in eosinophil-deficient ΔdblGATA mice

**Fig. 5:**
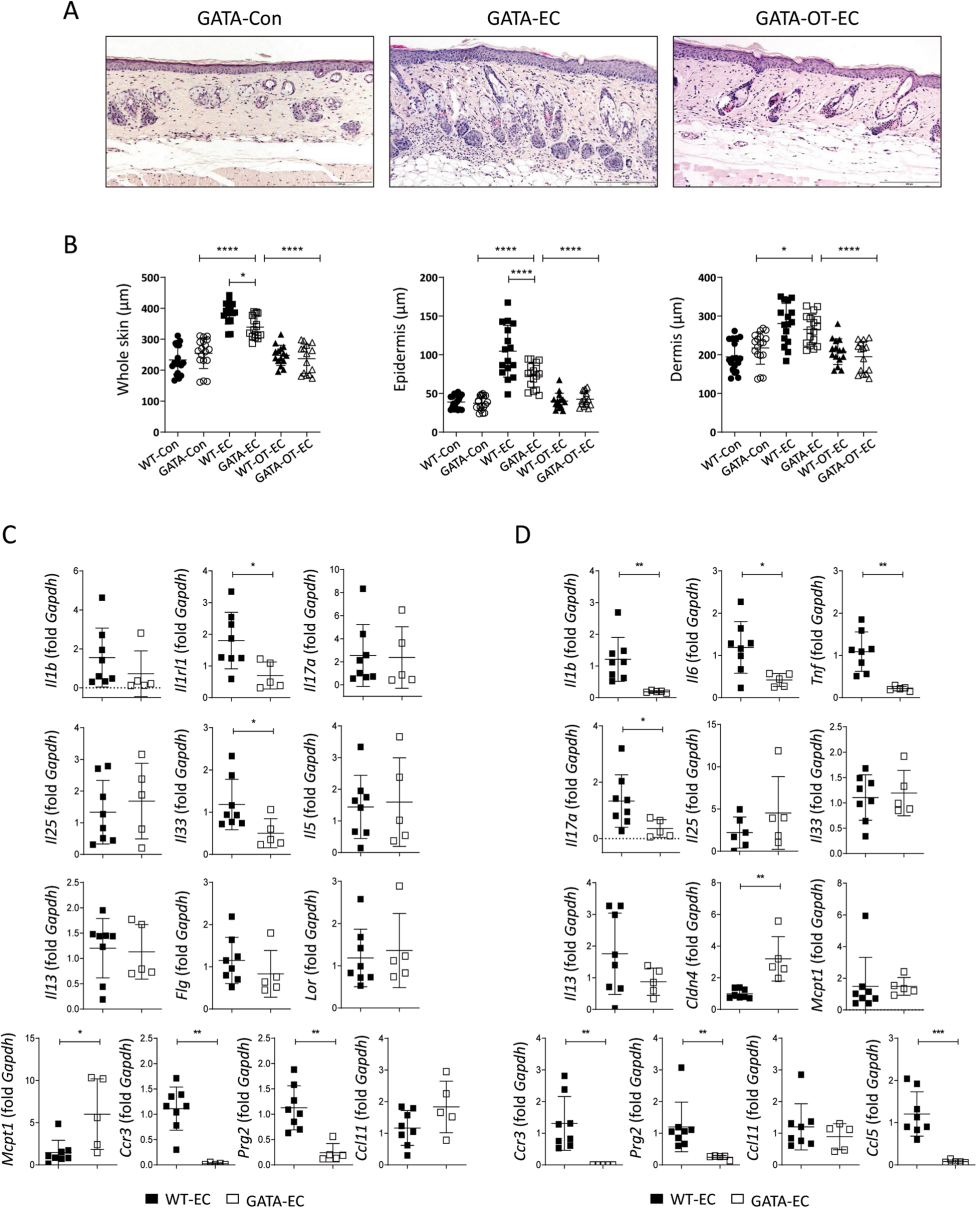
Inflammatory changes in the skin and small intestine elicited by EC challenge were attenuated in eosinophil-deficient ΔdblGATA mice. **A** Hematoxylin and eosin staining of skin from the control ΔdblGATA (GATA-Con), EC-challenged ΔdblGATA (GATA-EC), and orally tolerized and EC-challenged ΔdblGATA (GATA-OT-EC) mice. The images are representative of two independent experiments. The scale bar represents 200 μm. **B** The thickness of the whole skin (left), epidermis (middle), and dermis (right) of the indicated mice. The graphs show the mean ± SD values. *P < 0.05, ****P < 0.0001 (one-way ANOVA). **C** mRNA expression of *Il1b*, *Il1rl1*, *Il17a*, *Il25*, *Il33*, *Il5*, *Il13*, *Flg*, *Lor*, *Mcpt1*, *Ccr3*, *Prg2*, and *Ccl11* in the skin of the indicated mice. All data are representative of two independent experiments. The graphs show the mean ± SD values. *P < 0.05, **P < 0.01 (Student’s *t*-test for *Il1rl1* and *Il33*; Mann–Whitney tes*t* for *Mcpt1*, *Ccr3*, and *Prg2*). **D** mRNA expression of *Il1b*, *Il6*, *Tnf*, *Il17a*, *Il25*, *Il33*, *Il13*, *Cldn4*, *Mcpt1*, *Ccr3*, *Prg2*, *Ccl11*, and *Ccl5* in the small intestine of the indicated mice. All data are representative of two independent experiments. The graphs show the mean ± SD values. *P < 0.05, **P < 0.01, ***P < 0.001 (Student’s *t*-test for *Il1b*, *Il6*, *Tnf*, *Il17a*, and *Cldn4*; Mann–Whitney test for *Ccr3*, *Prg2*, and *Ccl5*).

As the induction of oral tolerance inhibited the EC challenge-induced increase in eosinophils in the small intestine, we hypothesized that eosinophils mediate inflammatory changes induced by the EC challenge in the small intestine. To confirm this, eosinophil lineage-ablated ΔdblGATA mice were subjected to EC challenge, and the immune responses in the skin and the small intestine were analyzed. Although eosinophils were not observed, Th2-type skin inflammation was successfully induced by the EC challenge, as demonstrated by a significant increase in skin thickness (Fig. 5A and B) and serum levels of anti-OVA IgE and IgG1 (Fig. S3), as well as by the upregulation of *Il5* and *Il13* in the skin of EC-challenged ΔdblGATA mice compared to that in the skin of control ΔdblGATA mice (Fig. S4A). However, the skin thickness and histological pathologic changes reduced markedly in EC-challenged ΔdblGATA mice compared to that in their wild type (WT) counterparts (Fig. 5A and B). In addition, the serum levels of OVA-specific IgE and IgG1 in the EC-challenged ΔdblGATA mice were lower than those in the EC-challenged WT mice (Fig. S3), indicating both local and systemic attenuation of allergic inflammatory responses with eosinophil deficiency. In line with these findings, the expression of genes associated with allergic inflammation, such as *Il1rl1* and *Il33*, decreased significantly in the skin of EC-challenged ΔdblGATA mice compared to that in their WT counterparts (Fig. 5C). In addition, the mRNA expression of inflammatory mediators, including *Il1b*, *Il6*, *Tnf*, *Il17a*, and *Ccl5*, decreased significantly in the small intestine, whereas *Cldn4* mRNA expression increased significantly in EC-challenged ΔdblGATA mice compared to that in EC-challenged WT mice (Fig. 5D). The mRNA expression of *Ccr3* and *Prg2*, the genes associated with the migration and activation of eosinophils, decreased significantly in both the skin and small intestine of EC-challenged ΔdblGATA mice compared to that in WT mice (Fig. 5C and D). It is unlikely that eosinophil deficiency influences oral tolerance induction, considering that the skin thickness and serum anti-OVA IgE and IgG1 levels in the orally tolerized and EC-challenged ΔdblGATA mice decreased significantly compared to that in EC-challenged ΔdblGATA mice and were comparable to those in orally tolerized and EC-challenged WT mice (Figs. 5B and S3).

### Inflammatory changes in the skin and small intestine of OVA EC-challenged mice were inhibited upon the antibiotic treatment-induced depletion of intestinal microbiota

**Fig. 6:**
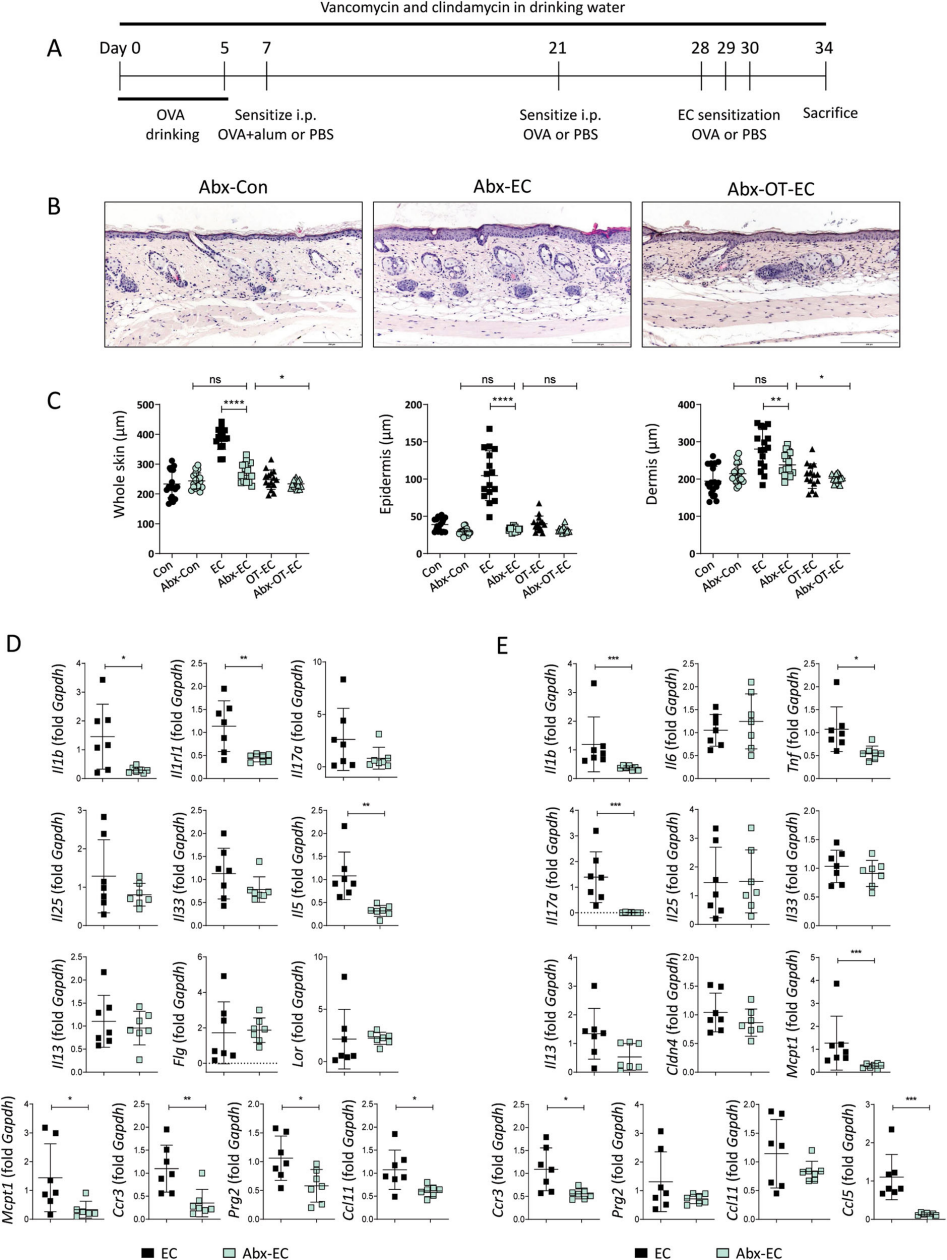
Inflammatory changes in the skin and small intestine elicited by EC challenge were attenuated in the antibiotic-treated mice. **A** Schedule of antibiotic treatments. **B** Hematoxylin and eosin staining of skin from the antibiotic-treated control (Abx-Con), antibiotic-treated and EC-challenged (Abx-EC), and antibiotic-treated, orally tolerized, and EC-challenged (Abx-OT-EC) mice. The images are representative of two independent experiments. The scale bar represents 200 μm. **C** Whole skin thickness (left), epidermal thickness (middle), and dermal thickness (right) of the indicated mice. The graphs show the mean ± SD values. *P < 0.05, **P < 0.01, ****P < 0.0001 (one-way ANOVA). **D** mRNA expression of *Il1b*, *Il1rl1*, *Il17a*, *Il25*, *Il33*, *Il5*, *Il13*, *Flg*, *Lor*, *Mcpt1*, *Ccr3*, *Prg2*, and *Ccl11* in the skin of the indicated mice. All data are representative of two independent experiments. The graphs show the mean ± SD values. *P < 0.05, **P < 0.01 (Student’s *t*-test for *Il1b*, *I1rl1*, *Il5*, *Prg2*, and *Ccl11*; Mann–Whi*t*ney test for *Mcpt1* and *Ccr3*). **E** mRNA expression of *Il1b*, *Il6*, *Tnf*, *Il17a*, *Il25*, *Il33*, *Il13*, *Cldn4*, *Mcpt1*, *Ccr3*, *Prg2*, *Ccl11*, *a*nd *Ccl5* in the small intestine of the indicated mice. All data are representative of two independent experiments. The graphs show the mean ± SD values. *P < 0.05, ***P < 0.001 (Student’s *t*-test for *Tnf*, *Ccr3*, and *Ccl5*; Mann–Whitney test for *Il1b*, *Il17a*, and *Mcpt1*).

We hypothesized that commensal intestinal microbiota might regulate immune responses in the skin and the small intestine, as the microbial composition was altered by EC challenge or oral tolerance. To explore this idea, we orally treated mice with a combination of vancomycin and clindamycin to deplete intestinal bacteria (Fig. 6A). Real-time polymerase chain reaction using cecal samples collected from antibiotic-treated mice revealed a significant reduction in gram-positive and gram-negative bacterial phyla (Fig. S5). Although the antibiotic-treated and EC-challenged mice showed a significant increase in serum anti-OVA IgE and anti-OVA IgG1 levels (Fig. S6) and an upregulation in the levels of skin inflammatory mediators compared to that observed in the antibiotic-treated control group (Fig. S7A), the histopathological changes in skin inflammation were almost completely inhibited compared to that in EC-challenged mice that did not undergo oral antibiotic treatment (Fig. 6B and C). Analysis of the skin revealed significantly decreased expression of the genes encoding innate (*Il1b* and *Il1rl1*) and Th2-type cytokines (*Il5*), as well as of the genes encoding proteins associated with the activation of eosinophils (*Ccr3*, *Prg2*, and *Ccl11*) and mast cells (*Mcpt1*) in antibiotic-treated and EC-challenged mice (Fig. 6D). Under antibiotic treatment, the expression of inflammatory cytokines in the small intestine was not enhanced upon the EC challenge (Fig. S7B), and we observed a significant decrease in the expression of *Il1b*, *Tnf*, *Il17a*, *Mcpt1*, *Ccr3*, and *Ccl5* in the small intestine of EC-challenged mice (Fig. 6E). The depletion of intestinal microbiota exerted insignificant effects on the induction of oral tolerance, as the antibiotic-treated, orally tolerized, and EC-challenged mice showed a significant reduction in dermal thickness (Fig. 6C) and serum anti-OVA IgE levels (Fig. S6), and the expression of allergic inflammatory mediators (Fig. S7A) in the skin also decreased compared to that in the antibiotic-treated and EC-challenged mice.

## Discussion

As the intestine is exposed to vast quantities of foreign antigenic materials and is colonized by multiple commensal microbes^16^, oral tolerance, the local and systemic immune unresponsiveness induced by orally ingested innocuous antigens, is critical for the maintenance of immune homeostasis^16,23^. We previously demonstrated that oral tolerance induction inhibits allergic inflammation in the skin induced by EC antigen exposure with the upregulation of tolerogenic immune mediators in the mesenteric lymph node^18^. It remains largely undetermined whether oral tolerance induces immunological changes in the small intestine, which provide a vast surface for the absorption of orally administered antigens and contains a large compartment of innate and adaptive immune cells^21^. Here, we demonstrated that a previously uncharacterized function of the small intestine contributes to AD pathogenesis and oral tolerance mediated protection against the development of AD-like inflammation by modulating intestinal immune microenvironments.

Epithelial barrier dysfunction of the skin, attributable to a combination of genetic and environmental factors, is not only a hallmark of AD but also a significant risk factor for the development of EC sensitization to food proteins^24^. In our study, a significant increase in the expression of genes encoding innate cytokines (*Il6*), Th17-type inflammatory cytokine (*Il17a*), and eosinophil-associated marker (*Ccr3*) and a decrease in the expression of genes encoding intestinal epithelial tight junction molecule (*Cldn4*) were observed in the small intestines of EC-challenged mice. Although the role of IL-17, a key cytokine implicated in autoimmune diseases, remains largely unresolved in allergic disease, recent studies have shown that Th17 immune responses contribute to chronic inflammation in AD^25,26^. As EC-challenged mice showed *Il17a* upregulation in their skin, although at insignificant levels, we suggest that IL-17 could be responsible for the pathogenesis of AD in both the skin and intestine. Considering that the expression of inflammatory mediators in the large intestine was not influenced by EC challenge (Fig. S8), it is plausible to suggest that cutaneous exposure to protein antigens through the damaged skin epithelium induces inflammatory changes and epithelial damage in the small intestine. In agreement with this idea, mechanical skin injury has been observed to increase intestinal permeability through the systemic release of inflammatory cytokines from damaged keratinocytes^27^. We also observed an increase, although insignificant, in the serum levels of soluble CD14, which reflects gut barrier dysfunction^28^, in EC-challenged mice, (*P* = 0.0745, Fig. S9). The absorption of nutrients only occurs in the small intestine because digestive enzymes and nutrient transporters are embedded in the epithelium of the small intestine^21^. In addition, compared to the lamina propria of colon, that of the small intestine is populated with multiple leukocytes, with a higher frequency of dendritic cells, Th17 cells, and eosinophils, which contribute to the maintenance of the small intestinal barrier integrity^29–31^. The induction of Foxp3^+^ regulatory T cells in the mesenteric lymph node is crucial for inducing systemic unresponsiveness in response to antigens introduced via the oral route^23,32^. We previously observed the expression of tolerogenic immune mediators and an increase in regulatory T cells in the mesenteric lymph nodes of mice protected from allergic skin inflammation via oral tolerance induction^18^. Although the expression of *Foxp3* mRNA in the small intestine did not change with oral tolerance in the EC-challenged mice (data not shown), oral tolerance led to the downregulation of the inflammatory mediators and eosinophil-associated markers and restored *Cldn4* expression in the small intestine with the alleviation of cutaneous allergic inflammatory features induced by EC exposure to OVA. Considering that the immune microenvironment in the small intestine provides signals to dendritic cells, which recognize and present orally administered antigens to naive T cells in the mesenteric lymph node^33^, we propose that oral tolerance promotes homeostatic immune responses in the small intestine and provides protection against cutaneous sensitization by inhibiting the sensitization of gut-associated lymphoid tissue to orally administered antigens.

The exposure of tape-stripped skin to OVA in sensitized mice increased the number of eosinophils in both the skin and small intestine, with increased mRNA expression of the markers associated with eosinophil infiltration and activation. Accordingly, compared to that in the WT mice, the pathogenic skin changes induced upon the EC challenge were attenuated in eosinophil-deficient mice, with a decrease in the levels of several inflammatory mediators in the skin and small intestine. In agreement with this finding, it has been recently demonstrated that EC allergen exposure leads to the local infiltration of eosinophils at the site of allergen challenge and also increases eosinophil concentration in the allergen non-exposed intestine^34^. Eosinophils are end-stage effector leukocytes implicated in the pathogenesis of Th2-type inflammation owing to their ability to release cytotoxic granules stored in their cytoplasm^35^. In addition to these cytotoxic proteins, eosinophils possess an array of preformed molecules associated with a wide range of biological responses in specific tissues where they are distributed^36,37^. Since the lamina propria of the small intestine has the largest pool of eosinophils in the body, immune mediators released by small intestinal eosinophils shape the microenvironments of the intestine in association with various homeostatic immune responses^37^. In eosinophil-deficient ΔdblGATA mice, EC challenge did not lead to an increase in the levels of small intestinal inflammatory mediators (Fig. S4B), and a significant decrease was observed in the expression of *Il1b*, *Il6*, *Tnf*, and *Ccl5*, along with an increase in the expression of *Cldn4* in the small intestine following EC exposure to OVA compared to that in WT mice. Considering that a decrease in eosinophil levels and the expression of the listed mediators was observed in the small intestine in orally tolerized and EC-challenged mice, we suggest that oral tolerance controls intestinal inflammatory changes induced by the skin antigen challenge by regulating inflammatory activation of eosinophils distributed in the small intestine.

As the primary interfaces with the external environment, the skin and intestine play essential roles in the maintenance of physiological homeostasis^19,38^. Although the specifics of the bidirectional communication between the skin and the intestine remain largely unknown, we have demonstrated that the EC OVA challenge induced a compositional change in the intestinal microflora, and that the antibiotic-mediated depletion of intestinal bacteria ameliorates the severity of experimental AD induced by EC challenge. The induction of oral tolerance to OVA prior to the EC challenge blocked the global shift in intestinal bacterial communities and the associated inflammatory changes in the skin and small intestine induced by the EC challenge, indicating that the intestinal microbiome plays a critical role in regulating host immune responses. As a dysregulated intestinal microbiota has been linked to the increased risk of AD^3,39^, we hypothesized that the depletion of intestinal bacteria before the induction of oral tolerance or EC challenge may increase susceptibility to experimental AD. However, mice with depleted intestinal microflora showed attenuated systemic and localized inflammatory responses, with a significant decrease in epidermal pathogenic changes. In line with this finding, psoriatic dermatitis was shown to be ameliorated in adult mice with depleted gut bacteria^40^. Considering that the perturbation of intestinal bacteria early in life promotes susceptibility to a wide range of inflammatory diseases^40–42^, it is plausible to suggest a distinct role of the intestinal microflora in shaping immune responses in childhood and adulthood.

In summary, through a detailed analysis of the immune responses in the skin and intestine, we revealed that the induction of oral tolerance protects mice from AD-like dermatitis and inhibits the increase in small intestinal eosinophils and dysregulated alterations in gut microflora. The importance of small intestinal eosinophils and intestinal bacteria in susceptibility to experimental AD was further substantiated by attenuated pro-inflammatory changes in the skin in eosinophil-deficient and intestinal bacteria-depleted mice. As the expression of small intestinal Th2-type cytokines under EC challenge was comparable in the eosinophil-deficient and bacteria-depleted mice compared to that in the WT and untreated mice, the small intestinal eosinophils or intestinal bacteria may contribute to the maintenance of intestinal homeostatic microenvironments rather than influencing allergic sensitization of the intestine. Considering that eosinophil-deficient and bacteria-depleted mice continued to exhibit oral tolerance-induced attenuation of AD-like inflammation, we speculate that additional regulatory subsets of the intestine contribute to the regulation of allergic skin inflammation, which needs to be elucidated further.

## Materials and methods

### Mice

Seven-to-ten-week-old female BALB/c mice (Orientbio, Gapyeng, Korea) and age- and sex-matched ΔdblGATA mice (Jackson Laboratory, Bar Harbor, ME, USA) were maintained under standard temperature and humidity in the specific pathogen-free facilities of the Center of Animal Care and Use of Lee Gil Ya Cancer and Diabetes Institute, Gachon University. All the experiments complied with the institutional principles for animal welfare.

### Oral tolerance induction and OVA skin challenge

To induce oral tolerance to OVA (Sigma-Aldrich, St. Louis, MO, USA), the mice were administered with 1% OVA (A5253, Grade II, Sigma-Aldrich) in drinking water provided ad libitum for 5 days, while a control group was provided with normal drinking water. Two days after the oral treatment, the mice were sensitized through intraperitoneal injection of 100 μg of OVA (A5503, Grade V, Sigma-Aldrich) and 4 mg of alum (Inject™ Alum, Thermo Scientific, Waltham, MA, USA). The control mice were sham-sensitized with phosphate-buffered saline (PBS). After 14 days of sensitization, the mice were challenged through intraperitoneal injection of 100 μg of OVA (Grade V, Sigma-Aldrich) or PBS. Seven days after the challenge, an EC challenge with OVA (Grade V, Sigma-Aldrich) was performed as described^18,34^.

### Antibiotic treatment

To deplete intestinal bacteria, the mice were fed with a mixture of 250 mg/L clindamycin (Samjin Pharm, Seoul, Korea) and 100 mg/L vancomycin (Korea United Pharm, Seoul, Korea) in drinking water from the oral tolerance induction until the end of the EC challenge. Clindamycin and vancomycin were chosen as they target gram-negative and gram-positive bacteria, respectively^43^.

### Histologic analysis

Tissue specimens were obtained from patched skin. The specimens were fixed in 10% buffered formalin (Duksan, Ansan, Korea) and embedded in paraffin. Multiple 4-μm sections were stained with hematoxylin and eosin and analyzed by using bright-field microscopy (Olympus, Tokyo, Japan). To analyze immune cell infiltration in the skin, sections were stained with anti-mouse CD3e polyclonal antibody (ab5690, Abcam, Cambridge, MA, USA), anti-mouse F4/80 monoclonal antibody (mAb) (#123101, BM8, BioLegend, San Diego, CA, USA), anti-mouse Gr-1 mAb (#550291, RB6-8C5, BD Biosciences, San Diego, CA, USA), and anti-mouse major basic protein mAb (MT-14.7, provided by Dr. James J. Lee; Mayo Clinic, Scottsdale, AZ, USA) overnight at 4 °C, followed by biotinylated anti-rabbit IgG (Vector Laboratories, Burlingame, CA, USA) or anti-rat IgG (Vector Laboratories) secondary antibody. The sections were then incubated with streptavidin-conjugated peroxidase (Vector Laboratories) and developed with 3,3′-diaminobenzidine substrate (Vector Laboratories). The immune cell infiltration was quantified^44^ using i-SOLUTION™ (IMT i‐Solution Inc, Vancouver, BC, Canada).

### Isolation of leukocytes from the small intestine

Small intestinal leukocytes were harvested as described^45,46^. Briefly, segments of the small intestine were incubated with FACS buffer (PBS containing 10% fetal bovine serum, 20 mM HEPES, 100 U/mL penicillin, 100 μg/mL streptomycin, 1 mM sodium pyruvate, and 10 mM EDTA) for 30 min at 37 °C to remove epithelial cells and were washed extensively with PBS. Small intestinal segments were digested with 2 mg/mL collagenase D (Roche, Manheim, Germany) and 50 μg/mL DNase I (Roche) in RPMI 1640/10% fetal bovine serum with continuous stirring at 37 °C for 30 min. EDTA was added (10 mM final), and the cell suspension was incubated for an additional 5 min at 37 °C. After washing, the cells were subjected to density-gradient centrifugation in 40%/75% Percoll. The cells harvested from the interface were washed and stained for flow cytometry.

### Flow cytometry

Fc receptors of the isolated small intestinal leukocytes were blocked with anti-mouse CD16/CD32 (2.4G2, BD Biosciences) for 15 min at 4 °C; the cells were stained for 30 min at 4 °C with the following antibodies: mAbs against CD45 (30-F11), CD4 (RM4-5), B220 (RA3-6B2), CD68 (FA-11), and CD11b (M1/70), purchased from Biolegend; anti-CD11C (HL3) and SiglecF (E50-2440) mAb, purchased from BD Biosciences; anti-CCR3 (83101) mAb obtained from R&D Systems (Minneapolis, MN, USA); and anti-CD8 (53-6.7) mAb, obtained from eBioscience (San Diego, CA, USA). Each sample was analyzed with a FACS Calibur™ flow cytometer (BD Biosciences) and the data were processed using FlowJo software (Tree Star, Ashland, OR, USA).

### Microbiota analysis

Genomic DNA was isolated from frozen stool samples using the QIAamp® Fast DNA Stool kit (Qiagen, Hilden, Germany) according to the manufacturer’s instructions. Bacterial DNA was amplified, targeting the V3–V4 regions of the 16S rRNA gene^47,48^. A subsequent amplification was performed to add multiplexing indices and the sequencing adapter. The amplified products were pooled and normalized using PicoGreen (Thermo Scientific). Sequencing was conducted at Macrogen Inc. (Seoul, Korea), using an Illumina MiSeq (Illumina, San Diego, CA, USA).

### Real-time polymerase chain reaction analysis

RNA was extracted from the skin and small intestine of the mice using QIAzol® lysis reagent (Qiagen) and subsequently column-purified with an RNeasy® Mini Kit (Qiagen). The RNA (500 ng) was treated with DNase I (New England Biolabs, Ipswich, MA, USA), and cDNA was synthesized using an iScript™ cDNA synthesis kit (Bio-Rad Laboratories, Hercules, CA, USA). The real-time polymerase chain reaction was performed using an iQ SYBR® Green Supermix (Bio-Rad Laboratories) with a CFX96™ Real-Time System (Bio-Rad Laboratories). The primers are detailed in Table S1.

### Statistical analysis

Experiments were performed in duplicate or triplicate, and two independent tests were performed for each assay except for microbiota sequencing. The sample size for each experiment was chosen based on published literature with a similar methodology. Two-group comparisons were performed with either a two-tailed unpaired Student’s *t*-test or Mann–Whitney test. Data differences between groups were examined for statistical significance using one-way ANOVA with the Tukey post hoc test or Kruskal–Wallis test with Dunn’s post hoc test. The normality of the data distribution was analyzed using the Kolmogorov–Smirnov test. A *P* value <0.05 was considered significant. GraphPad Prism 8 (GraphPad, San Diego, CA, USA) was used for the data analysis. There were no studies in which investigators were blinded.

## Supplementary information

Supplementary information

## References

[1] Blauvelt A, Hwang ST, Udey MC (2003). 11. Allergic and immunologic diseases of the skin. J. Allergy Clin. Immunol..

[2] Heratizadeh A, Wichmann K, Werfel T (2011). Food allergy and atopic dermatitis: how are they connected?. Curr. Allergy Asthma Rep..

[3] Song H, Yoo Y, Hwang J, Na YC, Kim HS (2016). Faecalibacterium prausnitzii subspecies-level dysbiosis in the human gut microbiome underlying atopic dermatitis. J. Allergy Clin. Immunol..

[4] Bin L, Leung DY (2016). Genetic and epigenetic studies of atopic dermatitis. Allergy Asthma Clin. Immunol..

[5] Egawa G, Kabashima K (2016). Multifactorial skin barrier deficiency and atopic dermatitis: Essential topics to prevent the atopic march. J. Allergy Clin. Immunol..

[6] Kantor R, Silverberg JI (2017). Environmental risk factors and their role in the management of atopic dermatitis. Expert Rev. Clin. Immunol..

[7] Brandt EB, Sivaprasad U (2011). Th2 Cytokines and Atopic Dermatitis. in. Cell. Immunol..

[8] Divekar R, Kita H (2015). Recent advances in epithelium-derived cytokines (IL-33, IL-25, and thymic stromal lymphopoietin) and allergic inflammation. Curr. Opin. Allergy Clin. Immunol..

[9] Wang LF, Lin JY, Hsieh KH, Lin RH (1996). Epicutaneous exposure of protein antigen induces a predominant Th2-like response with high IgE production in mice. J. Immunol..

[10] Spergel JM (1998). Epicutaneous sensitization with protein antigen induces localized allergic dermatitis and hyperresponsiveness to methacholine after single exposure to aerosolized antigen in mice. J. Clin. Invest..

[11] Sicherer SH, Sampson HA (1999). Food hypersensitivity and atopic dermatitis: pathophysiology, epidemiology, diagnosis, and management. J. Allergy Clin. Immunol..

[12] Lim NR, Lohman ME, Lio PA (2017). The role of elimination diets in atopic dermatitis-a comprehensive review. Pediatr. Dermatol..

[13] Pike MG, Heddle RJ, Boulton P, Turner MW, Atherton DJ (1986). Increased intestinal permeability in atopic eczema. J. Invest. Dermatol..

[14] Meresse B, Ripoche J, Heyman M, Cerf-Bensussan N (2009). Celiac disease: from oral tolerance to intestinal inflammation, autoimmunity and lymphomagenesis. Mucosal Immunol..

[15] Black PN (2005). Does atopy protect against enteric infections?. Allergy.

[16] Pabst O, Mowat AM (2012). Oral tolerance to food protein. Mucosal Immunol..

[17] Sakai T, Kogiso M, Mitsuya K, Komatsu T, Yamamoto S (2006). Defect of oral tolerance in NC/Nga mice. J. Med. Invest..

[18] Baek JO, Roh JY, Jung Y (2017). Oral tolerance inhibits atopic dermatitis-like type 2 inflammation in mice by modulating immune microenvironments. Allergy.

[19] Salem I, Ramser A, Isham N, Ghannoum MA (2018). The gut microbiome as a major regulator of the gut-skin axis. Front. Microbiol..

[20] Bernard M (2017). IL-1beta induces thymic stromal lymphopoietin and an atopic dermatitis-like phenotype in reconstructed healthy human epidermis. J. Pathol..

[21] Mowat AM, Agace WW (2014). Regional specialization within the intestinal immune system. Nat. Rev. Immunol..

[22] Schirmer M (2016). Linking the human gut microbiome to inflammatory cytokine production capacity. Cell.

[23] Weiner HL, da Cunha AP, Quintana F, Wu H (2011). Oral tolerance. Immunol. Rev..

[24] Tham EH, Rajakulendran M, Lee BW, Van Bever HPS (2020). Epicutaneous sensitization to food allergens in atopic dermatitis: what do we know?. Pediatr. Allergy Immunol..

[25] Czarnowicki T (2020). Evolution of pathologic T-cell subsets in patients with atopic dermatitis from infancy to adulthood. J. Allergy Clin. Immunol..

[26] Suarez-Farinas M (2013). Intrinsic atopic dermatitis shows similar TH2 and higher TH17 immune activation compared with extrinsic atopic dermatitis. J. Allergy Clin. Immunol..

[27] Leyva-Castillo JM (2019). Mechanical skin injury promotes food anaphylaxis by driving intestinal mast cell expansion. Immunity.

[28] Tabung FK (2017). Influence of dietary patterns on plasma soluble CD14, a surrogate marker of gut barrier dysfunction. Curr. Dev. Nutr.

[29] Carlens J (2009). Common gamma-chain-dependent signals confer selective survival of eosinophils in the murine small intestine. J. Immunol..

[30] Esplugues E (2011). Control of TH17 cells occurs in the small intestine. Nature.

[31] Mayer JU (2017). Different populations of CD11b(+) dendritic cells drive Th2 responses in the small intestine and colon. Nat. Commun..

[32] Ko HJ, Chang SY (2015). Regulation of intestinal immune system by dendritic cells. Immune Netw..

[33] Ruane DT, Lavelle EC (2011). The role of CD103(+) dendritic cells in the intestinal mucosal immune system. Front. Immunol..

[34] Olbrich CL (2020). Remote allergen exposure elicits eosinophil infiltration into allergen nonexposed mucosal organs and primes for allergic inflammation. Mucosal Immunol..

[35] Rothenberg ME, Hogan SP (2006). The eosinophil. Annu. Rev. Immunol..

[36] Kim HJ, Jung Y (2020). The emerging role of eosinophils as multifunctional leukocytes in health and disease. Immune Netw..

[37] Shah K, Ignacio A, McCoy KD, Harris NL (2020). The emerging roles of eosinophils in mucosal homeostasis. Mucosal Immunol.

[38] O’Neill CA, Monteleone G, McLaughlin JT, Paus R (2016). The gut-skin axis in health and disease: a paradigm with therapeutic implications. Bioessays.

[39] Lee MJ (2018). Perturbations of gut microbiome genes in infants with atopic dermatitis according to feeding type. J. Allergy Clin. Immunol..

[40] Zanvit P (2015). Antibiotics in neonatal life increase murine susceptibility to experimental psoriasis. Nat. Commun..

[41] Russell SL (2012). Early life antibiotic-driven changes in microbiota enhance susceptibility to allergic asthma. EMBO Rep..

[42] Baron R (2020). The relationship of prenatal antibiotic exposure and infant antibiotic administration with childhood allergies: a systematic review. BMC Pediatr..

[43] You JS (2019). Commensal-derived metabolites govern Vibrio cholerae pathogenesis in host intestine. Microbiome.

[44] Kang JK (2017). Increased intracellular Ca(2+) concentrations prevent membrane localization of PH domains through the formation of Ca(2+)-phosphoinositides. Proc. Natl Acad. Sci. U. S. A..

[45] Jung Y (2015). IL-1beta in eosinophil-mediated small intestinal homeostasis and IgA production. Mucosal Immunol..

[46] Roh J (2020). Functional expression of piezo1 in dorsal root ganglion (DRG) neurons. Int. J. Mol. Sci..

[47] Kim J (2019). Metagenomic analysis of isolation methods of a targeted microbe, *Campylobacter jejuni*, from chicken feces with high microbial contamination. Microbiome.

[48] Kim OH (2020). High-phytate/low-calcium diet is a risk factor for crystal nephropathies, renal phosphate wasting, and bone loss. Elife.

