# Supplementary material for: Small intestinal immune-environmental changes induced by oral tolerance inhibit experimental atopic dermatitis
Source: Cell Death Dis. 2021 Mar 4;12(3):243. doi: 10.1038/s41419-021-03534-w (PMC7933185; doi:10.1038/s41419-021-03534-w)
Supplement: Supplementary file 1 — Supplementary information [file 41419_2021_3534_MOESM1_ESM.docx]

**Supplementary information**

**1. Supplementary figures and figure legends**

**
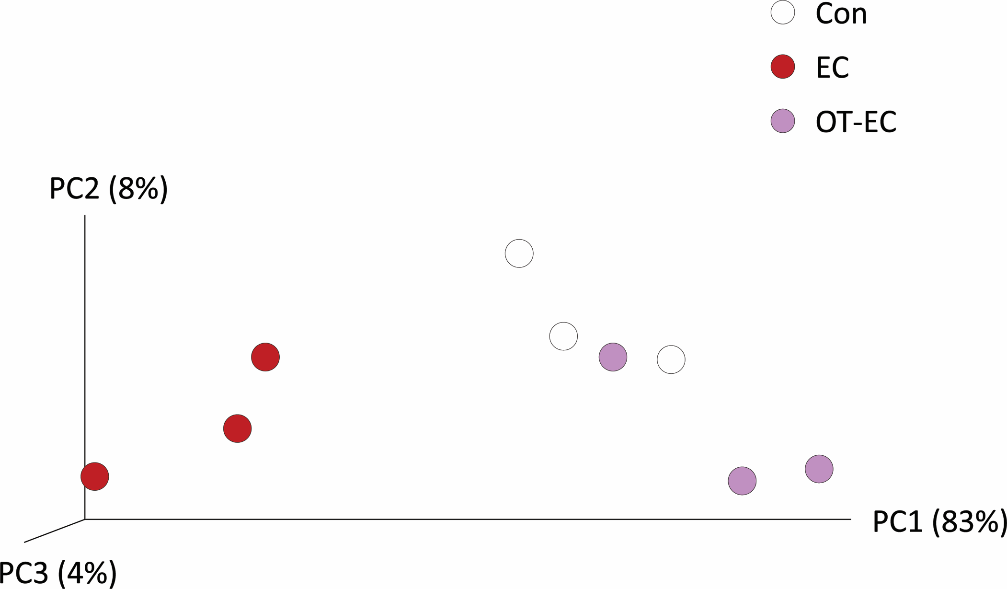
**

**Figure S1. Principal coordinate analysis (PCoA) 3D plot showing clustering of bacterial groups of cecal samples.** PCoA was performed using a weighted unifrac distance matrix. Each sample is represented by a point with the control (Con), EC-challenged (EC), and orally-tolerized and EC-challenged (OT-EC) mice.

**

**

**Figure S2. The number of small intestinal lamina propria cells.** Isolated small intestinal lamina propria (LP) cells from the indicated mice were counted. The graphs show the mean ± SD values.

**

**

**Figure S3. Serum levels of OVA-specific immunoglobulin in wild-type (WT) and ΔdblGATA (GATA) mice.** OVA-specific IgE (R35-118, BD Bioscience) and IgG1 (A85-1, BD Bioscience) titers in serum samples of the indicated mice were analyzed by enzyme-linked immunosorbent assay. All data are representative of two independent experiments (n = 6–10 mice per group). The graphs show the mean ± SD values. **P* < 0.05, ****P* < 0.001, *****P* < 0.0001 (Kruskal-Wallis test).

**

**

**Figure S4. Expression of inflammatory markers in the skin and small intestine of ΔdblGATA mice.** (A) mRNA expression of *Il1b*, *Il1rl*, *Il25*, *Il33*, *Il5*, *Il13*, *Flg*, *Lor*, *Mcpt1*, *Ccr3*, *Prg2*, and *Ccl11* in the skin of the indicated mice. All data are representative of two independent experiments. The graphs show the mean ± SD values. **P* < 0.05 (Kruskal-Wallis test). (B) mRNA expression of *Il1b*, *Il6,* *Tnf*, *Il25*, *Il33*, *Il13*, *Cldn4*, *Mcpt1*, *Ccr3*, *Prg2*, *Ccl11*, and *Ccl5* in the small intestine of the indicated mice. All data are representative of two independent experiments. The graphs show the mean ± SD values. **P* < 0.05 (Kruskal-Wallis test).

**
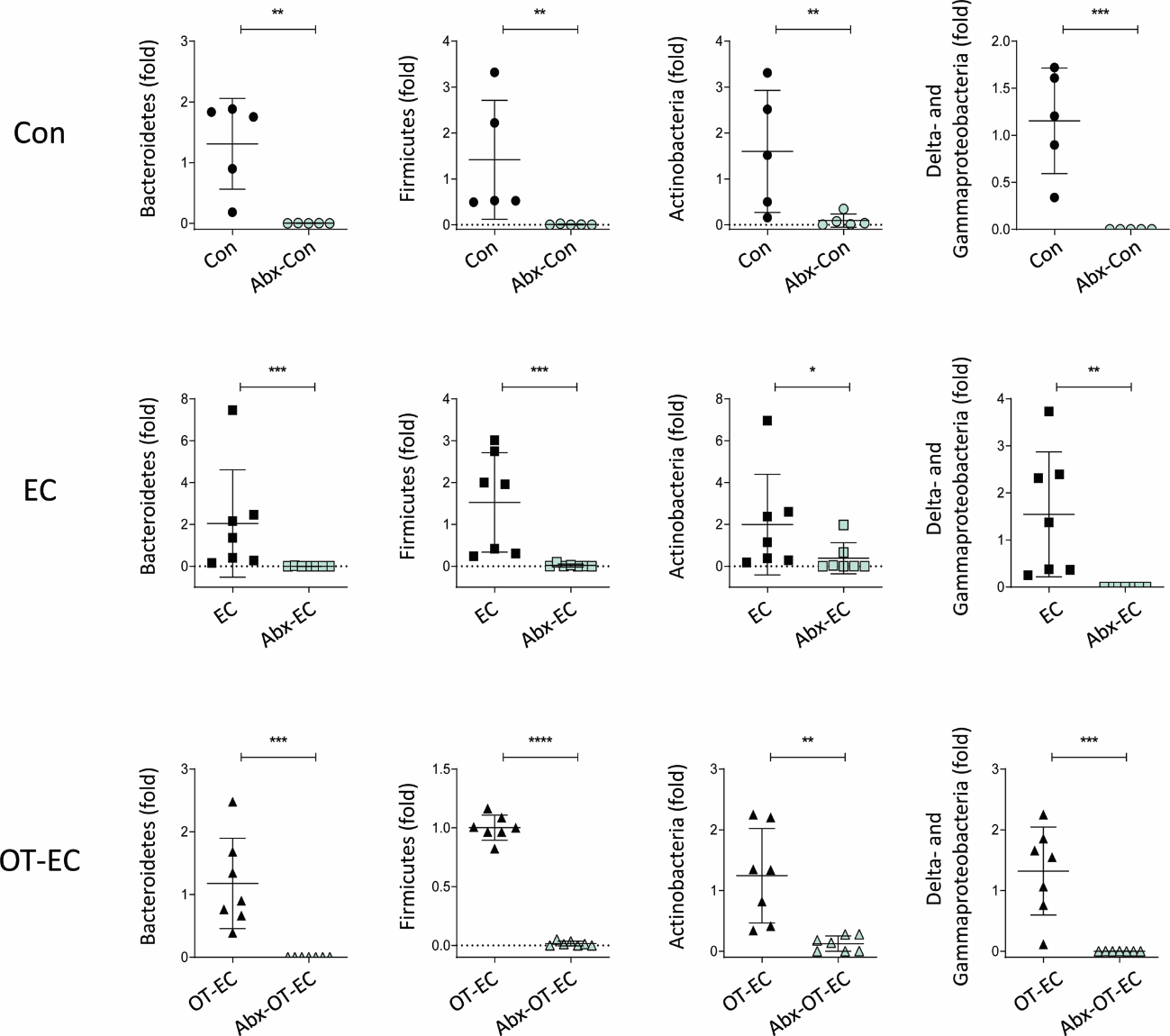
**

**Figure S5. Cecal bacteria phylum in the antibiotic-treated mice.** A real-time polymerase chain reaction of microbiota in the cecal contents of the control (Con), EC-challenged (EC), and orally-tolerized and EC-challenged (OT-EC) mice with or without antibiotics (Abx) treatment. The quantity of the 16S rRNA gene from each bacterial group was normalized to the quantity of the Eubacteria 16S rRNA gene. The graphs show the mean ± SD values. **P* < 0.05, ***P* < 0.01, ****P* < 0.001, *****P* < 0.0001 (Student’s *t*-test for Delta- and Gammaproteobacteria of the Con and EC mice, and all bacterial phyla of the OT-EC mice; Mann-Whitney test for Bacteroidetes, Firmicutes, and Actinobacteria of the Con and EC mice).

**

**

**Figure S6. Serum levels of OVA-specific immunoglobulin in the antibiotic-treated mice.** OVA-specific IgE and IgG1 titers in the sera of the indicated mice were analyzed using enzyme-linked immunosorbent assay. All data are representative of two independent experiments (n = 5–9 mice per group). The graphs show the mean ± SD values. **P* < 0.05, ***P* < 0.01, ****P* < 0.001 (Kruskal-Wallis test).

**
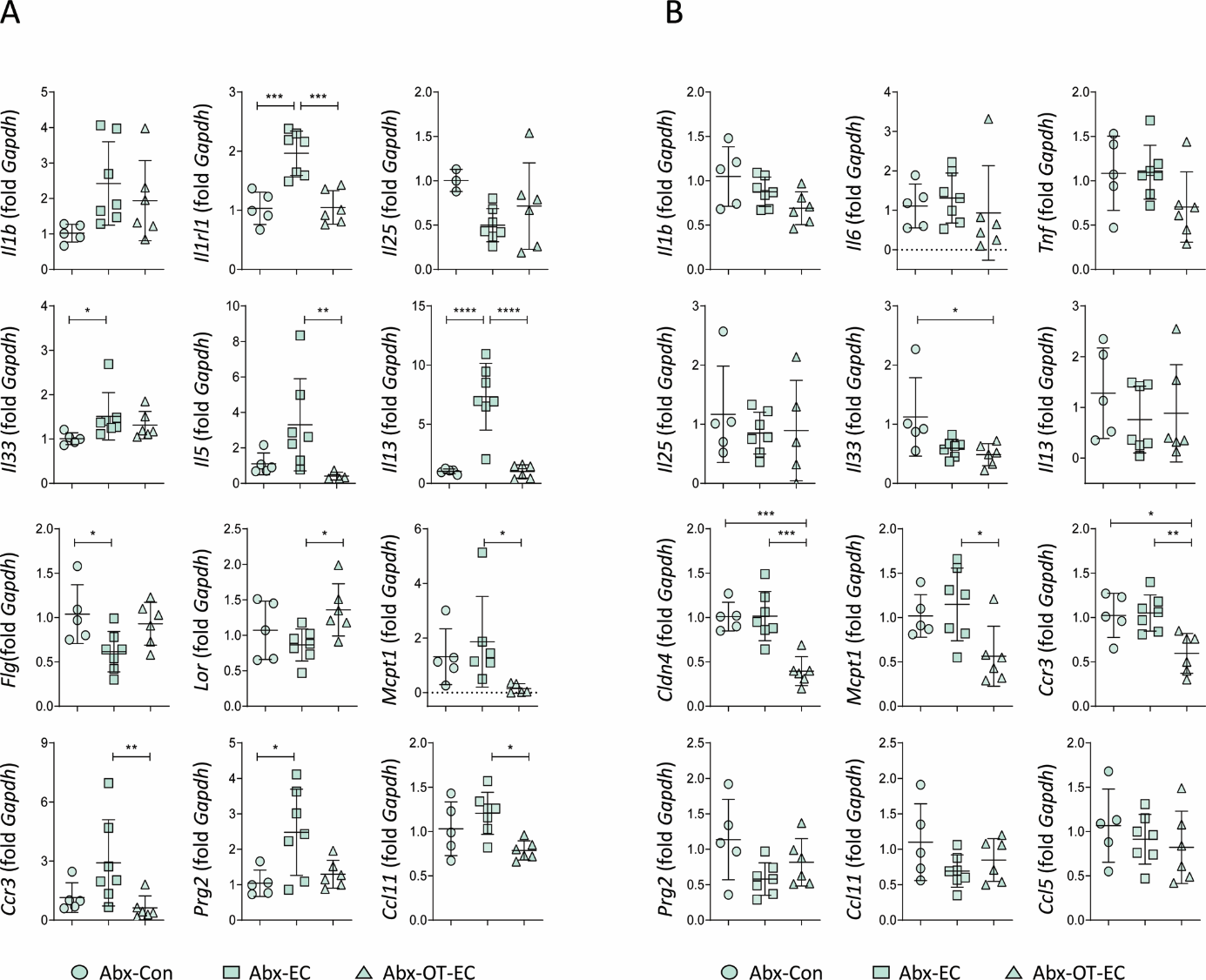
**

**Figure S7. Expression of inflammatory markers in the skin and small intestine of the antibiotic-treated mice.** (A) mRNA expression of *Il1b*, *Il1rl*, *Il25*, *Il33*, *Il5*, *Il13*, *Flg*, *Lor*, *Mcpt1*, *Ccr3*, *Prg2*, and *Ccl11* in the skin of the indicated mice. All data are representative of two independent experiments. The graphs show the mean ± SD values. **P* < 0.05, ***P* < 0.01, ****P* < 0.001, *****P* < 0.0001 (one-way ANOVA for *Il1rl1*, *Il13*, *Flg*, *Lor*, *Prg2*, and *Ccl11*; Kruskal-Wallis test for *Il33, Il5, Mcpt1*, and *Ccr3*). (B) mRNA expression of *Il1b*, *Il6*, *Tnf*, *Il25*, *Il33*, *Il13*, *Cldn4*, *Mcpt1*, *Ccr3*, *Prg2*, *Ccl11*, and *Ccl5* in the small intestine of the indicated mice. All data are representative of two independent experiments. The graphs show the mean ± SD values. **P* < 0.05, ***P* < 0.01, ****P* < 0.001 (one-way ANOVA for *Cldn4*, *Mcpt1*, and *Ccr3*; Kruskal-Wallis test for *Il33*).

**

**

**Figure S8. Expression of inflammatory markers in the large intestine.** mRNA expression of *Il1b*, *Il6,* *Tnf*, *Il25*, *Il33*, *Il13*, *Cldn4*, *Mcpt1*, *Ccr3*, *Prg2*, *Ccl11*, and *Ccl5* in the large intestine of the control (Con), EC-challenged (EC), and orally-tolerized and EC-challenged (OT-EC) mice. All data are representative of two independent experiments. The graphs show the mean ± SD values.

**

**

**Figure S9. Serum levels of soluble CD14 (sCD14).** sCD14 in serum samples of the indicated mice were analyzed using enzyme-linked immunosorbent assay (R&D Systems). The graphs show the mean ± SD values.

**2. Supplementary table**

**Table S1. Primer sequences for the real-time polymerase chain reaction**

| Target gene | Primer sequence |
| --- | --- |
| *Gapdh* | Forward: 5′- CTG GTA TGA CAA TGA ATA CGG -3′  Reverse: 5′- GCA GCG AAC TTT ATT GAT GG -3′ |
| *Il1b* | Forward: 5′- GCA ACT GTT CCT GAA CTC AAC T -3′  Reverse: 5′- ATC TTT TGG GGT CCG TCA ACT -3′ |
| *Il1rl1* | Forward: 5′- CAT TAC TAT CCT GTG CCA TT -3′  Reverse: 5′- CAT TCT CTG TCT GTC AAT CA -3′ |
| *Il17a* | Forward: 5′- GAC TTC CTC CAG AAT GTG AA-3′  Reverse: 5′- TGG AAC GGT TGA GGT AGT-3′ |
| *Il25* | Forward: 5′- TAT GAG TTG GAC AGG GAC TTG A -3′  Reverse: 5′- TGG TAA AGT GGG ACG GAG TTG -3′ |
| *Il33* | Forward: 5′- TCC AAC TCC AAG ATT TCC CCG -3′  Reverse: 5′- CAT GCA GTA GAC ATG GCA GAA -3′ |
| *Il5* | Forward: 5′- ACA AGC AAT GAG ACG ATG AG -3′  Reverse: 5′- CCA CGG ACA GTT TGA TTC TT -3′ |
| *Il13* | Forward: 5′- CCT GGC TCT TGC TTG CCT T -3′  Reverse: 5′- GGT CTT GTG TGA TGT TGC TCA -3′ |
| *Flg* | Forward:5’- AGG AGG GAG AGG AAG GAA A -3’  Reverse: 5′- GAA TCT TGT TGG TGT CTG TGT T -3’ |
| *Lor* | Forward: 5′- ACA TCA GCA TCA CCT CCT TC -3’  Reverse: 5′- TCT TTC CAC AAC CCA CAG G -3’ |
| *Mcpt1* | Forward: 5′- ACA CTC CCG TCC TTA CAT -3′  Reverse: 5′- GAT TCA CTC TTG CTC ACA TC -3′ |
| *Ccr3* | Forward: 5′- CAG TGC TTT TGG GTG TTT GTC -3′  Reverse: 5′- GAT TTC TAG GGT CTG TGT GCC -3′ |
| *Prg2* | Forward: 5′- TGA AAC TTC TGA CTC CAA AAG CC -3′  Reverse: 5′- CGG CAT TAG CTC TTC CCC T -3′ |
| *Ccl11* | Forward: 5′- AGC TAG TCG GGA GAG CCT AC -3′  Reverse: 5′- AAG GAA GTG ACC GTG AGC AG -3′ |
| *Il6* | Forward: 5′- TAG TCC TTC CTA CCC CAA TT -3′  Reverse: 5′- TTG GTC CTT AGC CAC TCC TTC -3′ |
| *Tnf* | Forward: 5′- CCT GTA GCC CAC GTC GTA G -3′  Reverse: 5′- GGG AGT AGA CAA GGT ACA ACC C -3′ |
| *Cldn4* | Forward: 5′- TGG ATG GAC GGG TTT GAG -3′  Reverse: 5′- CAC ACT GGG CTG CTT CTA -3′ |
| *Ccl5* | Forward: 5′- TGC TCC AAT CTT GCA GTC GT -3′  Reverse: 5′- TCT TCT CTG GGT TGG CAC AC -3′ |
| Eubacteria | Forward: 5′- AAA CTC AAA KGA ATT GAC GG -3′  Reverse: 5′- CTC ACR RCA CGA GCT GAC -3′ |
| Bacteroidetes | Forward: 5′- GTT TAA TTC GAT GAT ACG CGA G -3′  Reverse: 5′- TTA ASC CGA CAC CTC ACG G -3′ |
| Firmicutes | Forward: 5′- GGA GYA TGT GGT TTA ATT CGA AGC A -3′  Reverse: 5′- AGC TGA CGA CAA CCA TGC AC -3′ |
| Actinobacteria | Forward: 5′- TGT AGC GGT GGA ATG CGC -3′  Reverse: 5′- AAT TAA GCC ACA TGC TCC GCT -3′ |
| Deata- and Gammaproteobacteria | Forward: 5′- GCT AAC GCA TTA AGT RYC CCG -3′  Reverse: 5′- GCC ATG CRG CAC CTG TCT -3′ |

* Nucleotide symbols: R = A or G; Y = C or T; N = any nucleotide; W = A or T; M = A or C; K = T or G; S = C or G; and H = A/C/T^1^.

**Reference**

1. Yang, Y. W., Chen, M. K., Yang, B. Y., Huang, X. J., Zhang, X. R., He, L. Q. et al. Use of 16S rRNA Gene-Targeted Group-Specific Primers for Real-Time PCR Analysis of Predominant Bacteria in Mouse Feces. *Appl. Environ. Microbiol.* **81,** 6749-6756 (2015).
